# Critical dialogue: how imaging evaluates and guides pelvic exenteration surgery—a multidisciplinary perspective

**DOI:** 10.1186/s13244-025-02201-4

**Published:** 2026-02-16

**Authors:** Stephanie Nougaret, Verity Wood, Tamara Glyn, Quentin Denost, Damian Tolan, Doenja M. J. Lambregts

**Affiliations:** 1 Department of Radiology, Montpellier Cancer Institute, Montpellier, France; 2 PINKCC lab, U1194, Montpellier Research Cancer Institute, University of Montpellier, Montpellier, France; 3 Te Whatu Ora Waitaha, Christchurch, New Zealand; 4 Department of Surgery, Waitemata District Health Board, Auckland, New Zealand; 5 Department of Surgery and critical care, University of Otago, Christchurch, New Zealand; 6 Department of Surgery, Bordeaux Colorectal Institute, Bordeaux, France; 7 Department of Radiology, Leeds Teaching Hospitals NHS Trust, Leeds, UK; 8 Department of Radiology, The Netherlands Cancer Institute, Amsterdam, The Netherlands; 9 GROW Research, Institute for Oncology and Reproduction—University of Maastricht, Maastricht, The Netherlands

## Current practice and knowledge

Pelvic exenteration (PE) is one of the most radical procedures in oncologic surgery, performed for locally advanced or recurrent pelvic malignancies involving colorectal, gynecologic, urologic, or musculoskeletal tumors [1–3]. The objective is the achievement of microscopically negative margins (R0), which remains the single most important prognostic factor [2]. Historically associated with high perioperative mortality and significant morbidity, advances in surgical technique, perioperative care, and patient selection have led to continuous improvement in exenteration R0 rates without increasing major surgical complication or morbidity [4]. Nevertheless, PE continues to carry substantial functional consequences, underscoring the need for precise preoperative assessment and planning to carefully balance the likelihood of attaining an R0 resection against the potential long-term functional deficits [5].

Conventional surgical resections, such as total mesorectal excision, follow embryological tissue planes [3]. In contrast, exenterative surgery frequently demands dissection across multiple pelvic compartments, often disrupted due to prior surgery, removal of adjacent organs, and sometimes resection of bone, vessels, or nerves [1]. This complexity necessitates more advanced multimodality imaging and a more detailed radiological reporting than is typically required for routine planning of less advanced tumors. Magnetic resonance imaging (MRI) has become the cornerstone for local pelvic assessment in PE, offering unparalleled soft tissue resolution and anatomical detail. Computed tomography (CT) provides complementary information for distant staging and can aid in vascular assessment, while positron emission tomography (PET/CT) helps in detecting multifocal disease, lymph node involvement, and extrapelvic metastatic spread [6, 7].

A recent international collaborative effort from ESGAR, SAR, ESUR, and the PelvEx group has led to the development of the first structured imaging guidelines for PE [7]. These guidelines stress the importance of standardized MRI protocols, structured reporting, and the use of anatomical compartments as a framework for surgical planning [7]. The goal is to reduce variability in imaging quality and reporting, and to ensure that key details that influence surgical decision-making are consistently and accurately captured.

## Advancements and new developments

Technical improvements in MRI have substantially transformed preoperative assessment. High-resolution T2-weighted imaging in three orthogonal planes remains essential to visualize the lesion and its relationship with neighboring structures. Three-dimensional isotropic imaging and contrast-enhanced three-dimensional short inversion time inversion-recovery (STIR) fast spin-echo sequences may be particularly useful for nerve assessment [8, 9]. Diffusion-weighted imaging and post-contrast sequences add functional detail, helping to distinguish viable tumor from fibrosis resulting from previous surgery, or when assessing tumor response following chemoradiotherapy [10, 11]. PET/CT serves as a valuable adjunct for metabolic characterization and detection of nodal or anatomically occult disease sites. Accurate tumor delineation in these settings relies on integrated interpretation of anatomic and functional imaging, underscoring the importance of multidisciplinary collaboration between radiologists and nuclear medicine specialists.

Equally transformative has been the move toward standardized and anatomically oriented reporting, instead of relying on traditional TNM-based staging. Current practice in PE emphasizes a compartment-based approach to pelvic anatomy. Rather than just reporting disease selectively organ by organ, radiologists now define involvement of the structures within the central, anterior, posterior, and lateral compartments [12, 13]. The BONVUE checklist (bones, organs, nerves, vessels, ureters, and extra tumor sites) provides a systematic and reproducible method for reporting.

- Bones: assessment of cortical erosion, marrow invasion, or sacral and pelvic sidewall involvement.
- Organs: detailed evaluation of visceral invasion, including bladder, rectum, uterus, vagina, prostate, or bowel loops.
- Nerves: identification of tumor contact or encasement of the lumbosacral plexus, sciatic nerve, or obturator nerve, which may critically impact resectability.
- Vessels: evaluation of arterial and venous involvement, including encasement, narrowing, or occlusion of external/internal iliac vessels.
- Ureters: assessment of ureteric encasement.
- Extra-tumor sites: detection of distant pelvic deposits, peritoneal disease, lymph nodes, or unsuspected extra-pelvic spread.

This framework provides surgeons with a structured roadmap aligned with operative planes and anticipated resections.

Artificial intelligence (AI) is emerging as a potentially powerful complement in this setting. Although not yet implemented into standard reporting practice, natural language generation tools are being explored to standardize reports, reduce inter-observer variability, and ensure critical surgical details are consistently addressed [14–17]. Additionally, automated segmentations of pelvic structures (e.g., vessels, bones, and ureters) along with AI-assisted tumor mapping and 3D reconstructions are already being piloted [18–20]. These tools can offer surgeons enhanced visualization of tumor extension and its relationship to resection planes. Additionally, this may extend into the operating theater, where augmented reality platforms fuse preoperative MRI with the surgical field or robotic systems, guiding dissections with unprecedented precision [21, 22].

Collectively, these technical and conceptual advances have redefined the radiologist’s role in pelvic oncology. No longer confined to descriptive reporting, radiologists are now integral members of multidisciplinary tumor boards, presenting annotated images, highlighting threatened margins, and anticipating reconstructive challenges. In specialized PE centers, radiology reports have evolved into explicit surgical roadmaps, forming the basis of operability assessments and surgical strategy. The ongoing integration of advanced imaging with AI-enabled visualization promises to strengthen this role further, embedding radiology at the heart of surgical decision-making in complex pelvic cancer care.

## How imaging guides treatment planning

The decision whether and how to proceed with PE hinges on whether an R0 resection can be achieved. High-quality MRI is indispensable in this regard, defining the relationship between the tumor and critical structures such as the sacrum, pelvic sidewall, iliac vessels, and sacral nerve roots. Disease that abuts or invades these structures requires highly specialized surgery, and in some cases define the limit of operability. For example, posterior disease involving the presacral fascia may necessitate sacrectomy or sub-periosteal excision, while lateral disease encroaching on the iliac vessels or sciatic nerve roots requires extended lateral sidewall clearance or may even preclude surgery [23–25]. Anterior extension can involve the bladder, uterus, vagina, or prostate, with the need for cystectomy, vaginectomy, or prostatectomy depending on the structures involved [2].

Imaging also plays a critical role in reconstructive planning. Assessment of vascular anatomy is critical when planning flap reconstructions, while delineation of urinary tract involvement informs decisions regarding preservation or diversion [26, 27]. In this way, imaging not only dictates the extent of resection but also anticipates functional outcomes and quality of life implications. Post-treatment MRI plays an equally important role by helping to distinguish tumor regression from fibrosis, guiding decisions about whether to pursue aggressive surgery after neoadjuvant therapy [10, 28].

When patients are deemed inoperable based on imaging—either due to unresectable sidewall involvement, high sacral extension, or encasement of major vascular structures—management typically shifts toward non-surgical approaches. In this context, MRI not only establishes the limits of operability but also provides critical information for alternative strategies. Radiation oncologists increasingly rely on high-quality pelvic imaging to define target volumes for re-irradiation, boost delivery, or advanced modalities such as intensity-modulated radiotherapy (IMRT) and stereotactic body radiotherapy (SBRT) [29–31]. Functional sequences, including diffusion-weighted imaging, can help characterize viable disease and guide dose painting to the most aggressive subregions [32].

Ultimately, imaging serves a dual role: for operable patients, it provides the surgical roadmap; for inoperable cases, it guides radiation planning and palliative strategies. This reinforces the central position of radiology in multidisciplinary care, ensuring that treatment pathways—whether surgical or non-surgical—are grounded in precise anatomical and functional assessment.

In summary, PE exemplifies the essential synergy between radiology and surgery. Modern imaging not only defines who is eligible for this radical procedure but also shapes the surgical plan, informs reconstruction, and anticipates outcomes. Radiology has evolved from passive interpretation to active integration, shifting from description to direct influence in clinical decision-making. By further aligning imaging practices with surgical needs, we can improve patient selection, maximize R0 resection rates, and reduce unnecessary morbidity, ultimately offering patients with advanced pelvic malignancies a real chance at long-term survival.

## Data Availability

Not applicable.
